# Development and Validation of a Prognostic Nomogram Based on the Systemic Immune-Inflammation Index for Resectable Gallbladder Cancer to Predict Survival and Chemotherapy Benefit

**DOI:** 10.3389/fonc.2021.692647

**Published:** 2021-06-29

**Authors:** Lin Li, Tai Ren, Ke Liu, Mao-Lan Li, Ya-Jun Geng, Yang Yang, Huai-Feng Li, Xue-Chuan Li, Run-Fa Bao, Yi-Jun Shu, Hao Weng, Wei Gong, Wan Yee Lau, Xiang-Song Wu, Ying-Bin Liu

**Affiliations:** ^1^ Department of Biliary-Pancreatic Surgery, Renji Hospital Affiliated to Shanghai Jiao Tong University School of Medicine, Shanghai, China; ^2^ Shanghai Key Laboratory of Biliary Tract Disease, Renji Hospital, Shanghai, China; ^3^ Shanghai Research Center of Biliary Tract Disease, Renji Hospital, Shanghai, China; ^4^ Department of General Surgery, Xinhua Hospital, Affiliated to Shanghai Jiao Tong University School of Medicine, Shanghai, China; ^5^ Faculty of Medicine, The Chinese University of Hong Kong, Shatin, New Territories, Hong Kong, Hong Kong; ^6^ Shanghai Cancer Institute, State Key Laboratory of Oncogenes and Related Genes, Shanghai, China

**Keywords:** gallbladder carcinoma, nomogram, systemic immune-inflammation index, chemotherapy, prognostic marker

## Abstract

**Objectives:**

To investigate the prognostic significance of the systemic immune-inflammation index (SII) in patients after radical cholecystectomy for gallbladder cancer (GBC) using overall survival (OS) as the primary outcome measure.

**Methods:**

Based on data from a multi-institutional registry of patients with GBC, significant prognostic factors after radical cholecystectomy were identified by multivariate Cox proportional hazards model. A novel staging system was established, visualized as a nomogram. The response to adjuvant chemotherapy was compared between patients in different subgroups according to the novel staging system.

**Results:**

Of the 1072 GBC patients enrolled, 691 was randomly selected in the discovery cohort and 381 in the validation cohort. SII>510 was found to be an independent predictor of OS (hazard ratio [HR] 1.90, 95% confidence interval [CI] 1.42-2.54). Carbohydrate antigen 199(CA19-9), tumor differentiation, T stage, N stage, margin status and SII were involved in the nomogram. The nomogram showed a superior prediction compared with models without SII (1-, 3-, 5-year integrated discrimination improvement (IDI):2.4%, 4.1%, 5.4%, P<0.001), and compared to TNM staging system (1-, 3-, 5-year integrated discrimination improvement (IDI):5.9%, 10.4%, 12.2%, P<0.001). The C-index of the nomogram in predicting OS was 0.735 (95% CI 0.683-0.766). The novel staging system based on the nomogram showed good discriminative ability for patients with T2 or T3 staging and with negative lymph nodes after R0 resection. Adjuvant chemotherapy offered significant survival benefits to these patients with poor prognosis.

**Conclusions:**

SII was an independent predictor of OS in patients after radical cholecystectomy for GBC. The new staging system identified subgroups of patients with T2 or T3 GBC with negative lymph nodes who benefited from adjuvant chemotherapy.

**Clinical Trial Registration:**

ClinicalTrials.gov, identifier (NCT04140552).

## Introduction

Gallbladder cancer (GBC) is a highly malignant tumor that accounts for 80%-95% of biliary tract malignancies (1). Its relatively low incidence and indistinct symptomatology result in most patients presenting with an advanced disease at the time of diagnosis, thus, contributing to its dismal prognosis (2). American Joint Committee on Cancer(AJCC; eighth version) TNM Staging System is the most widely used system for GBC (3). However, survival outcomes vary widely for patients even within the same stage in this system, as multiple factors affect long-term survival of an individual patient (4, 5). Recently, accumulating evidences have suggested that the inflammation pathway is closely related to tumor development and progression (6–8). Inflammatory indices such as neutrophil-to-lymphocyte ratio (NLR), platelet-to-lymphocyte ratio (PLR) and Glasgow prognostic score (GPS) have been proven to have prognostic values for GBC (9–11). On the other hand, the systemic immune-inflammation index (SII), which is based on neutrophil, lymphocyte and platelet counts as first described in hepatocarcinoma cancer (12), has rarely been studied in GBC (13).

Nomogram, as a predictive statistical model for individual patients (14), have been shown to demonstrate advantages over the traditional staging systems in predicting long-term survival outcomes of patients (15–19). Many nomograms have been proposed as a practical tool to guide treatment for cancer (17, 20).

This study aimed to compare the prognostic value of several inflammatory indices, and to develop a nomogram by combining preoperative examinations and clinicopathological factors. Moreover, whether adjuvant therapy is necessary for patients after R0 radical cholecystectomy with negative regional lymph nodes is still controversial. The American Hepato-Pancreato-Biliary Association (AHPBA) consensus recommends adjuvant therapy for patients with stage II GBC or higher, but whether adjuvant chemotherapy or chemoradiation should be given is unknown (21). The National Comprehensive Cancer Network (NCCN, 2021 V.2) guideline suggests that GBC patients with negative lymph nodes after radical cholecystectomy should be followed-up with observation, or should receive the treatment regimens consisting of either fluoropyrimidine chemoradiation or chemotherapy with fluoropyrimidine or gemcitabine (22). To address this issue, a novel staging system was established in this study by integrating multiple clinicopathological factors to stratify T2 or T3 staging patients with negative lymph nodes and R0 resection margins to investigate whether adjuvant therapy was beneficial for these patients with unfavorable prognosis.

## Methods

### Patients

The Chinese Research Group of Gallbladder Cancer (CRGGC) is a multi-institutional registry cohort that retrospectively collected medical records of GBC patients in China, with a standardized protocol detailed in (23). This study enrolled consecutive patients who underwent radical cholecystectomy between January 2002 and January 2019 in 35 tertiary medical centers in China from the CRGGC. The inclusion criteria were as following (1): pathologically diagnosed GBC according to International Classification of Disease for Oncology (ICD-O-3); (2) data containing detailed preoperative blood results; (3) radical cholecystectomy performed for GBC. The exclusion criteria were patients with: (1) unknown T staging; (2) M1 staging or T4 staging, according to the AJCC 8^th^ staging system; (3) Tis or T1a staging after simple cholecystectomy; (4) clinical evidence of infection or other inflammatory conditions; (5) overall survival (OS) of less than 3 months, potentially due to postoperative complications (**Supplementary Figure 1**). Patients enrolled in the study were randomly selected into the validation cohort until the number of event had reached 200 to achieve an ideal sample size for validation (24).

To verify the response to adjuvant chemotherapy of different subgroups in the novel staging system, a different cohort of patients who underwent radical cholecystectomy between January 2008 and June 2019 at Renji Hospital Affiliated to Shanghai Jiao Tong University School of Medicine and Xinhua Hospital Affiliated to Shanghai Jiao Tong University School of Medicine were retrospectively studied. The selection criteria for inclusion were patients: (1) with AJCC 8^th^T2 or T3 GBC; (2) with specific information of adjuvant chemotherapy. The exclusion criteria were patients with: (1) histologically confirmed positive lymph nodes; (2) chemotherapeutic agents beyond the NCCN 2021 V.2 guideline; (3) long-term oral administration of Traditional Chinese Medicine after surgery; (4) OS of less than 3 months.

### Data Collection

Available pre-operative laboratory examination within a week before the data of surgery were collected. Results of pre-operative laboratory examination were identified from medical records, including: total bilirubin, CA19-9, absolute neutrophil, absolute lymphocyte, platelet, and absolute monocyte counts. The inflammatory indices were defined as neutrophil-to-lymphocyte ratio (NLR, absolute neutrophil count divided by absolute lymphocyte count), platelet-to-lymphocyte ratio (PLR, absolute platelet count divided by absolute lymphocyte count), lymphocyte-to-monocyte ratio (LMR, absolute lymphocyte count divided by absolute monocyte count), and SII (platelet count times NLR).

Overall survival (OS) was calculated from the date of surgery to the date of death or last follow-up, whichever came last. This study was censored on June 2020. Pathologic staging was done following the AJCC 8^th^ Staging System. For each variable, we required duplicated entry by two trained professionals. If any discrepancies were found, a third specialist would be brought in for discussion and make a final decision.

### Statistical Analysis

Continuous variables were transformed into categorical variables based on routine cutoffs in clinical application. Parameters such as NLR and SII were grouped as high and low by optimal cut-off points using the cut p function (R package survMisc).

Continuous data were compared using the unpaired t test, and categorical data using the chi-square test or Fisher’s exact test. Ordinal categorical variables were compared by Wilcoxon rank sum test. OS was examined by the Kaplan-Meier method and compared using the log-rank test. The associations of bilirubin and CA19-9 with SII levels were estimated using the Spearman rank-correlation coefficient.

Univariable Cox proportional hazards models were applied to select covariates with a significance level <0.05 into the following multivariate model. Harrell’s concordance index (C-index) was calculated for each model. Patients with missing data for covariates of interest were excluded.

Multivariable Cox regression was applied to establish a prediction model, then visualized by a nomogram. The final prediction model was selected by the backward stepdown selection process with the Akaike information criterion (AIC). The predictive accuracy and discriminative ability were determined by C-index and calibration curve, and assessed by comparing the nomogram-predicted against observed survival on application of bootstrapping with 1000 resamples. The integrated discrimination improvement (IDI) and decision curve analysis (DCA) was used to evaluate the predictive performance of different models. The total point of each patient in the validation cohort was calculated according to the established nomogram, and then Cox regression was performed for this cohort using the total points as a variate. Each patient was then assigned a score based on the nomogram, and the cut-off points were calculated using the spline curve.

All tests were two-sided, and P values of less than 0.05 were considered statistically significant. All statistical analyses were performed using the software R version 3.6.1.

## Results

### Clinicopathological Characteristics

Of the 1072 patients included in this study, 691 patients (64.5%) were randomly assigned into the discovery cohort, and 381 (35.5%) in the validation cohort. The median OS for the discovery and validation cohorts were 40.4 months (95% confidence interval [CI], 32.0-51.4 months) and 41.4 months (95% CI, 31.9-53.5 months), respectively. The corresponding median follow-up times for the 2 cohorts were 53.8 (range 3 months to 18 years) and 52.9 months (range 3 months to 12.6 years). The baseline clinicopathological characteristics were summarized in **Table 1**.

**Table 1:** Clinicopathological Characteristics of the GBC Patients in the Discovery and Validation Cohorts.

	Discovery cohort (N=691)	Validation cohort (N=381)	P value
Age^†^	62(57-69.5)	63(57-70)	0.48
Sex			0.68
Male	270	144	
Female	421	237	
CA19-9			0.96
≤40 U/ml	350	192	
>40 U/m	221	122	
NA	120	67	
Surgical approach			0.78
RC	405	240	
ERC	38	23	
LC+RC	127	67	
NA	121	51	
Total bilirubin			0.96
≤35 μmol/L	553	311	
>35 μmol/L	108	60	
NA	30	10	
Margin status			0.30
R0	65	27	
R1	567	334	
Rx	59	20	
Pathological type			0.97
ADC	569	310	
ADSC	35	13	
PADC	27	16	
NEC	10	7	
Other	50	35	
Tumor differentiation			0.96
Low	145	76	
Low to medium	118	68	
Medium	256	130	
Medium to high	40	23	
High	62	34	
T stage^‡^			0.17
T1	91	52	
T2	74	55	
T3	526	274	
N stage^‡^			0.62
N0	304	164	
N1	159	85	
N2	41	22	
Nx	187	110	
Microvascular invasion			0.64
Yes	68	31	
No	550	308	
NA	73	42	
Perineural invasion			0.43
Yes	118	54	
No	519	294	
NA	54	33	
Platelets			0.02
≤300*10^^9^/L	591	306	
>300*10^^9^/L	100	75	
NLR			0.14
≤2.3	331	165	
>2.3	360	216	
LMR			0.22
≤10	400	206	
>10	291	175	
PLR			0.56
≤144	370	197	
>144	321	184	
SII			0.40
≤510	343	179	
>510	348	202	

^†^Age is presented as the median (first quartile-third quartile).

^‡^T stage and N stage was classified according to the AJCC 8^th^ edition staging system.

ADC, adenocarcinoma; ADSC, adenosquamous carcinoma;PADC, papillary adenocarcinoma; NEC, neuroendocrine carcinoma; LC, laparoscopic cholecystectomy; RC, radical cholecystectomy; ERC, extended radical cholecystectomy; NLR, neutrophil-to-lymphocyte ratio; PLR, platelet-to-lymphocyte ratio; LMR, lymphocyte-to-monocyte ratio; SII, systemic immune-inflammation index.

### Discovery Cohort

The optimal cut-offs were 510 for SII, 2.3 for NLR, 144 for PLR, and 10 for LMR. The following predictors were associated with worse OS: age, CA19-9>40 U/ml, total bilirubin>35 µmol/L, more advanced surgical approach, R1 resection margin status, pathological type, poor tumor differentiation, microvascular invasion, perineural invasion, advanced T staging, advanced N staging, PLR>144, NLR>2.3, LMR>10, and SII>510 (**Table 2**). Multivariate analysis without preoperative inflammatory indices showed following predictors still associated with worse OS with statistical significance: CA19-9 levels, R1 resection margin status, poor tumor differentiation, and advanced T and N staging (**Table 2**).

**Table 2:** Cox Proportional Hazards Regression Models for Predictor Selection and Model Building.

	Univariate Analysis		Multivariate Analysis		Selected Factors for Building the Model	
	HR (95% CI)	P value	HR (95% CI)	P value	HR (95% CI)	P value
Age						
>60 versus ≤60 years	1.32 (1.06-1.64)	0.01	1.31 (0.92-1.86)	0.13	NA
Sex						
Female versus Male	0.95 (0.77-1.17)	0.63	NA	NA
CA19-9						
>40 versus ≤40 U/ml	2.19 (1.74-2.76)	<0.001	1. 59 (1.08-2.34)	0.01	1.67 (1.25-2.24)	<0.001
Total bilirubin						
>35 versus ≤35 µmol/L	2.00 (1.55-2.58)	<0.001	1.32 (0.83-2.10)	0.22	NA
Surgical approach						
ERC versus RC	1.76 (1.20-2.61)	0.003	1.69 (0.92-3.10)	0.08		
LC+RC versus RC	0.97 (0.74-1.28)	0.87	1.16 (0.70-1.91)	0.56		
Margin status						
R1 versus R0	2.63 (1.94-3.57)	<0.001	2.10 (1.19-3.68)	0.009	1.55 (1.01-2.39)	0.04
Pathological type						
ADSC versus ADC	1.23 (0.77-1.96)	0.37	1.05 (0.51-2.14)	0.88	NA
PADC versus ADC	0.24 (0.10-0.59)	0.001	0.45 (0.05-3.57)	0.45	
NEC versus ADC	1.60 (0.75-3.39)	0.21	4.61 (0.84-25.21)	0.07	
Else versus ADC	1.14(0.78-1.67)	0.48	1.20 (0.54-2.66)	0.64	
Tumor differentiation						
Low to medium versus Low	0.84 (0.62-1.15)	0.28	0.59 (0.35-1.01)	0.054	0.63 (0.42-0.96)	0.03
Medium versus Low	0.61 (0.47-0.80)	<0.001	0.53 (0.33-0.82)	0.005	0.53 (0.37-0.75)	<0.001
Medium to high versus Low	0.41 (0.24-0.70)	0.001	0.43 (0.18-1.04)	0.06	0.42 (0.21-0.84)	0.01
High versus Low	0.25 (0.15-0.42)	<0.001	0.38 (0.17-0.85)	0.01	0.40 (0.19-0.83)	0.01
T stage						
T2 versus T1	2.08 (1.25-3.47)	0.004	1.71 (0.70-4.12)	0.23	1.39 (0.66-2.92)	0.38
T3 versus T1	3.10 (2.05-4.67)	<0.001	2.71 (1.18-6.24)	0. 01	2.58 (1.31-5.10)	0.006
N stage						
N1 versus N0	1.80 (1.38-2.35)	<0.001	1.14 (0.72-1.80)	0.56	1.11 (0.77-1.59)	0.56
N2 versus N0	3.34 (2.26-4.94)	<0.001	2.84 (1.50-5.39)	0.001	2.43 (1.42-4.17)	0.001
Nx^†^ versus N0	1.53 (1.18-1.98)	0.001	1.42 (0.88-2.28)	0.14	1.40 (0.97-2.01)	0.06
Microvascular invasion						
Yes versus No	2.12 (1.55-2.90)	<0.001	1.73 (0.97-3.08)	0.06		
Perineural invasion						
Yes vs No	1.76 (1.35-2.28)	<0.001	1.13 (0.69-1.85)	0.61		
Platelets						
>300 versus ≤300×10^9^/L	1.19 (0.89-1.60)	0.31	NA		
LMR						
>10 versus ≤10	1.39 (1.13-1.71)	0.001	NA		
NLR						
>2.3 versus ≤2.3	1.97 (1.59-2.44)	<0.001	NA		
PLR						
>144 versus ≤144	1.58 (1.28-1.94)	<0.001	NA		
SII						
>510 versus ≤510	1.99 (1.61-2.45)	<0.001	NA	1.90(1.42-2.54)	<0.001

^†^Nx means that the N stage could not be judged from the pathological record or the surgeon did not obtain a sufficient number of lymph nodes.

HR, hazard ratio; CI, confidence interval; LC, laparoscopic cholecystectomy; RC, radical cholecystectomy; ERC, extended radical cholecystectomy; NA, not applicable.

As NLR, PLR, LMR and SII were predictors of OS on univariate analysis, multivariate models were compared to find out which index to include for further multivariate analysis (**Supplementary Table 1**). SII had the lowest AIC when compared to NLR (1249 versus 1255, log likelihood ratio test P value<0.001); PLR (1249 versus 1263, log likelihood ratio test P value<0.001) and LMR (1249 versus 1262, log likelihood ratio test P value<0.001). Moreover, SII had the highest C-index (0.735, compared to 0.732 for PLR, 0.732 for NLR and 0.722 for LMR). Indeed, all these models showed a higher C-index and lower AIC than the base model. In addition, the platelet count, which was included in SII but not in NLR, had a hazard ratio (HR) >1 (1.19, P=0.31). Based on these findings, SII was included for subsequent analyses.

Patients’ clinicopathologic characteristics categorized by high and low SII are summarized in **Supplementary Table 2**. Patients with high SII had significantly higher CA19-9 levels, total bilirubin and platelets levels and had more advanced tumor progression including T stage, microvascular invasion, nerve invasion and poorer tumor differentiation.

### Subgroup Analyses Showed the Effect of SII Depended on Obstructive Jaundice, CA19-9 Levels and Microvascular Invasion

**Figure 1:**
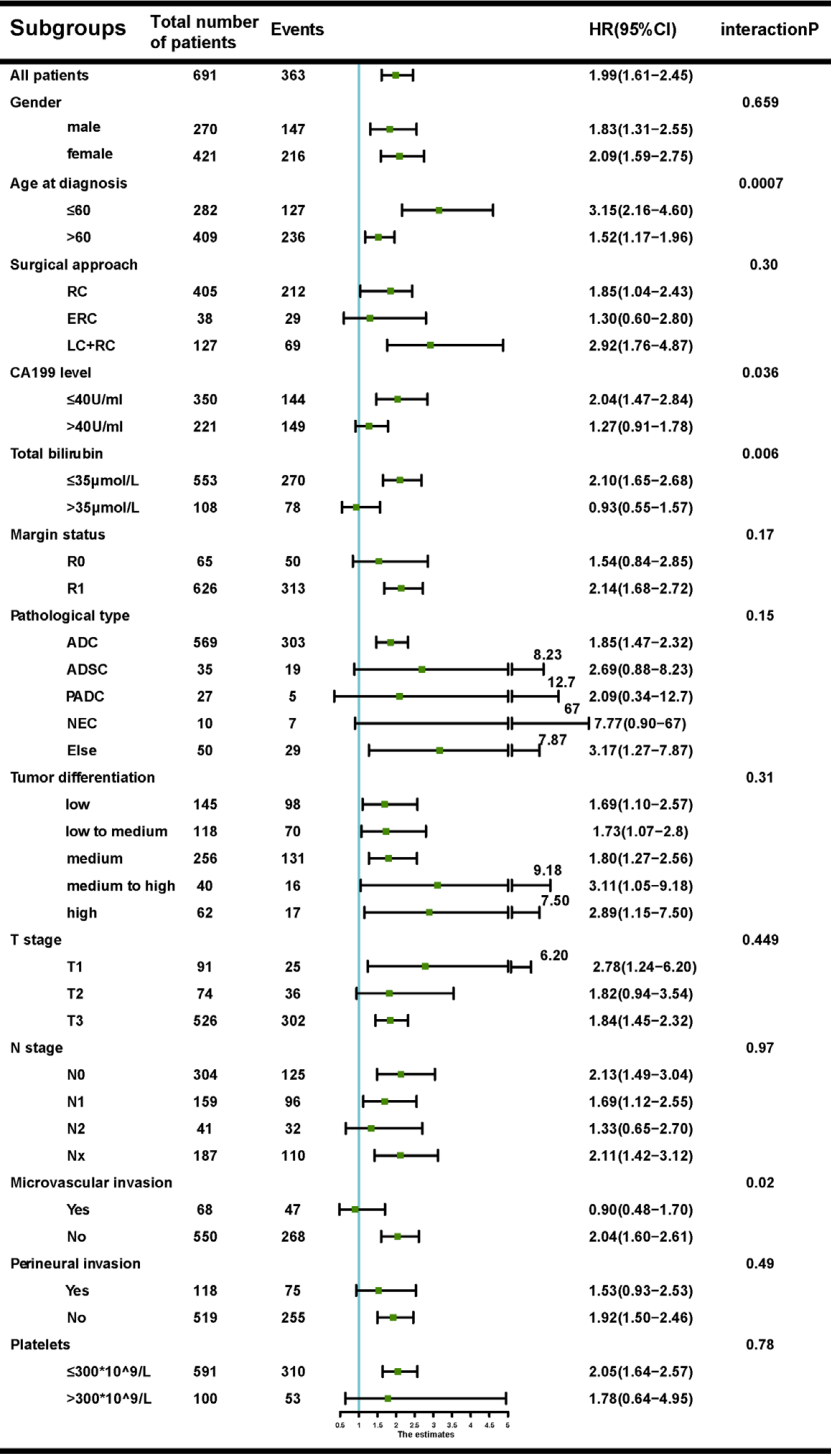
Forest plot of the association between systemic immune-inflammation index (SII) and overall survival (OS), according to different subgroups.

The associations between SII and OS were found in the discovery (**Supplementary Figure 2A**), validation (**Supplementary Figure 2B**) and total cohorts (**Supplementary Figure 2C**). Aziz et al. (25) reported that SII lost its prognostic ability with increase in bilirubin. Subgroup analyses showed no interaction between SII with any factors with the exception of bilirubin, CA19-9 levels and microvascular invasion. Significant associations were observed between SII and bilirubin levels (P=0.006), CA19-9 levels (P=0.03) or microvascular invasion(P=0.02) (**Figure 1**).

Of the 661 patients in the discovery cohort with available data on preoperative bilirubin levels, 108 presented with obstructive jaundice. Patients with SII<510 had significantly lower mean bilirubin levels than those with SII>510 (30.1μmol/L vs 58.5μmol/L, P<0.001). SII was correlated with pre-operative bilirubin levels (r_s_ =0.19, P<0.001). The associations between OS and SII in the settings of low (<35 µmol/L) and high (>35 µmol/L) bilirubin levels are shown in **Supplementary Figures 3A, D**. Similar results were observed in the validation cohort (**Supplementary Figures 3B, E**).

CA19-9 levels were found having similar association with the prognostic ability of SII. Patients with SII<510 had significantly lower mean CA19-9 levels than those with SII>510 (145.6 U/ml versus 238.1 U/ml, P=0.04). SII was correlated with CA19-9 levels (r_s_ =0.21, P<0.001). The prognostic ability of SII was then examined in different CA19-9 groups (≤40 U/ml versus >40 U/ml). We observed that the prognostic role of SII did not persist at high CA19-9 levels in the discovery (**Supplementary Figures 4A, D**), and validation cohort (**Supplementary Figures 4B, E**)

The prognostic ability of SII was also examined in patients with or without microvascular invasion in the discovery (**Supplementary Figures 5A, D**) and validation cohort (**Supplementary Figures 5B, E**). Patients with SII>510 were more likely to have microvascular invasion (P<0.001) and SII seemed to lose its prognostic value in patients with microvascular invasion.

### Prognostic Nomogram for OS

**Figure 2:**
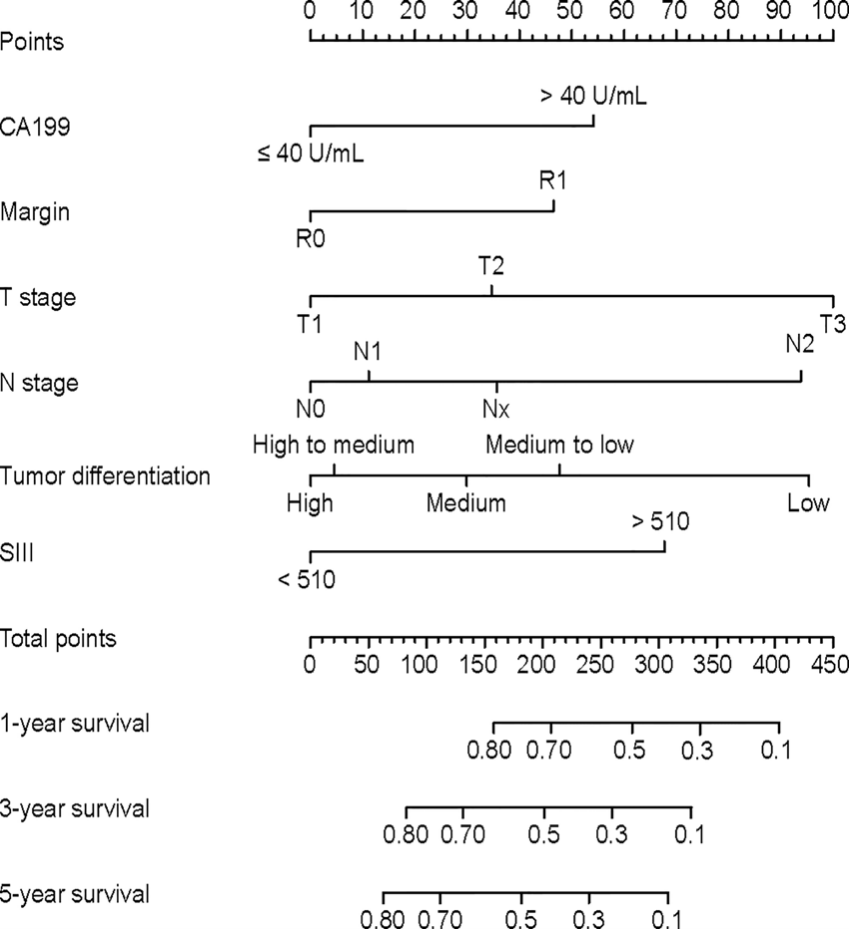
Nomogram for predicting overall survival in GBC patients.

As we observed an interaction between CA19-9 and SII (p for interaction <0.05), the interaction term was included into multivariable Cox analysis. The HR of SII*CA19-9 was 0.88 (95% CI 0.50-1.55), with a P value=0.67 (**Supplementary Table 1**). A nomogram was then established (**Figure 2**). The nomogram showed that T staging and tumor differentiation shared the largest contributions to prognosis, followed by N staging and SII. CA19-9 levels and resection margin status had moderate impact on prognosis.

The calibration curve plots showed agreement between nomogram predictions and actual observations in the discovery cohort, and acceptable consistency in the validation cohort (**Supplementary Figure 6**). In the discovery cohort, Harrell’s C-index for the nomogram in predicting OS (0.735, 95% CI, 0.697-0.782) was significantly higher than that for the base model without SII (0.726, 95% CI, 0.683-0.773, P<0.01) and higher than the AJCC 8^th^ TNM prediction (0.639, 95% CI, 0.601-0.676, p<0.01, P<0.01). In the validation cohort, the C-index was also significantly higher for the nomogram (0.686, 95% CI, 0.633-0.738) than the base model (0.674, 95% CI, 0.621-0.727, P<0.01) and higher than the AJCC 8^th^ TNM prediction (0.608, 95% CI, 0.568-0.647, p<0.01, P<0.01). The IDIs of the nomogram comparing with the multivariate model without SII were 2.4%, 4.1% and 5.4% for 1-,3-, and 5- year OS in the discovery cohort and 3.3%, 3.6%, 3.8% in the validation cohort, respectively (**Supplementary Figure 7**), Compared to the TNM staging system, the nomogram also showed better accuracy in estimating OS (IDI: 5.9%, 10.4%, 12.2% for 1,3,5 years OS in the discovery cohort and 6.6%, 7.9%, 7.6% in the validation cohort, data not shown). The DCA plot also indicated the nomogram was superior to the model without SII and TNM staging system (**Supplementary Figure 8**).

### Construction of the Novel Staging System

**Figure 3:**
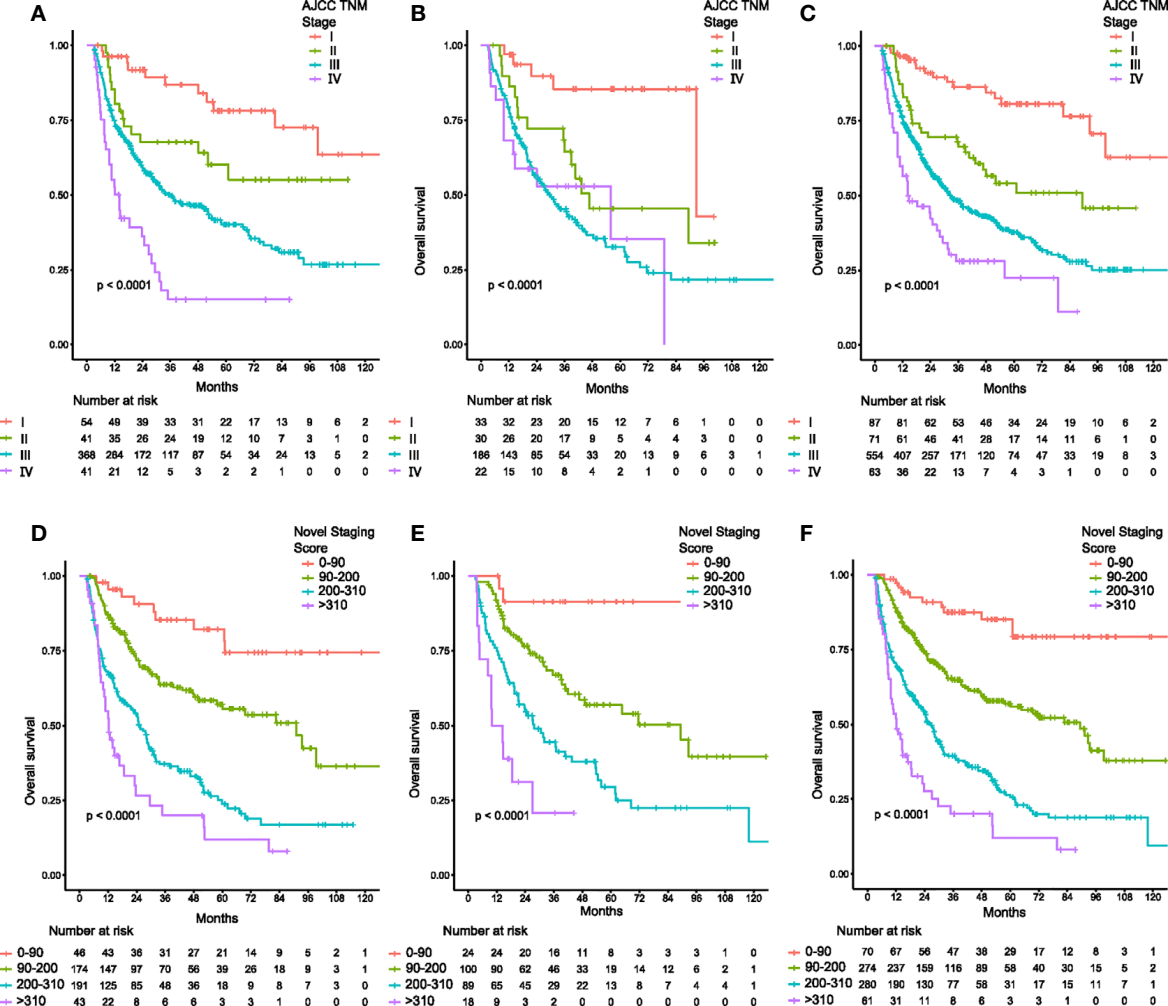
Kaplan-Meier curves of overall survival for patients classified by TNM staging system and the novel staging system. **(A)** discovery cohort classified by TNM system, **(B)** validation cohort classified by TNM system, **(C)** total cohort classified by TNM system, and **(D)** discovery cohort classified by the novel staging system, **(E)** validation cohort classified by the novel staging system, **(F)** total cohort classified by the novel staging system.

Based on the nomogram, an individual predictive score of OS for each patient can be estimated. When patients with scores of 0-90, 90-200, 200-310, and ≥310 were classified into stages I (n=70), II (n=274), II (n=280) and IV (n=61), respectively (**Figure 3F**), the groups showed distinct separation on long-term prognoses. The median OS was not calculable for stage I patients due to insufficient follow up time. However, for stage II, Ш and IV patients, the median OS were 82.6, 26.9 and 12.2 months, respectively. Of the 775 patients with TNM staging, the number of patients in TNM stages of I, II, Ш and IV were 87, 71, 554 and 63, respectively (**Figure 3C**). The discriminating ability of the nomogram was superior to the AJCC TNM staging system in the discovery (**Figures 3A, D**), and validation cohort (**Figures 3B, E**).

### Association Between the Nomogram and Survival Benefit From Adjuvant Chemotherapy in Patients With T2N0 or T3N0 GBC

The patients with T2N0 and T3N0 GBC who underwent R0 radical cholecystectomy in the total cohort were stratified using the nomogram to study whether there were subgroups of patients with significantly worse long-term survival. For patients with T2N0 GBC after radical cholecystectomy, the nomogram stratified these patients into separate groups with distinguished prognosis, with 5-year OS rates for stages I, II, Ш being 74%, 36%, and 33% (P=0.018, **Supplementary Figure 9A**), respectively. For patients with T3N0 GBC after radical cholecystectomy, the 5-year overall survival rates for stages II, Ш, IV were 65%, 24% and 11%, respectively (P<0.01, **Supplementary Figure 9B**).

**Figure 4:**
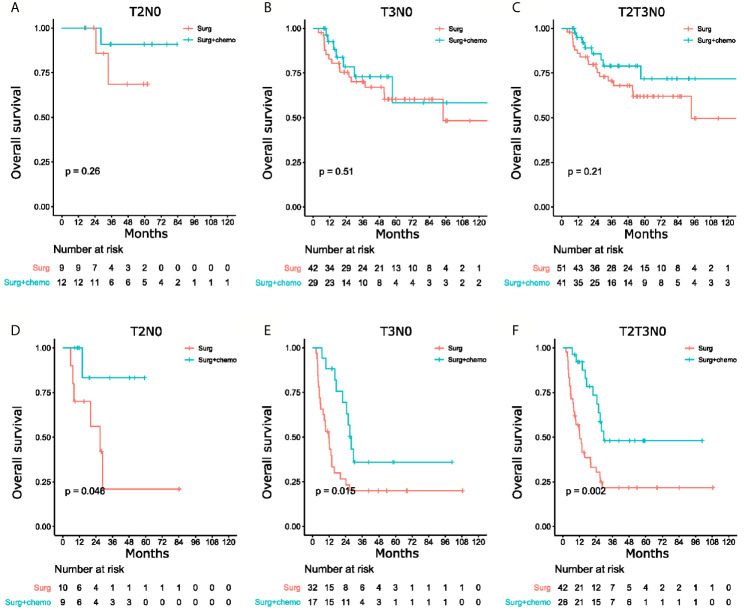
Kaplan-Meier curves of overall survival for patients with surgery only versus surgery and chemotherapy. **(A)** T2N0 patients with good prognosis, **(B)** T3N0 patients with good prognosis, **(C)** T2N0 and T3N0 patients with good prognosis, and **(D)** T2N0 patients with poor prognosis, **(E)** T3N0 patients with poor prognosis, **(F)** T2N0 and T3N0 patients with poor prognosis.

Till now, there is no definitive conclusion whether postoperative adjuvant therapy is necessary for T2N0 or T3N0 patients after radical cholecystectomy. As the novel staging system resulted in good stratification of these patients, this system was hypothesized to be able to identify patients who could benefit from adjuvant chemotherapy. To verify this hypothesis, another cohort of patients (**Supplementary Table 3**) who underwent radical cholecystectomy with or without adjuvant chemotherapy were enrolled. The chemotherapy regimens used between the two groups of patients were similar (P=0.97). Adjuvant chemotherapy provided significant survival benefit in this group of patients with T2N0 and T3N0 GBC who were known to have poor prognosis after radical cholecystectomy (**Figures 4D–F**), when compared to patients with good prognosis (**Figures 4A–C**). These results indicated the novel staging system was able to identify patients with unfavorable prognosis who could benefit from adjuvant chemotherapy.

## Discussion

Mounting evidences have accumulated to support that inflammatory biomarker plays an important role in prognostication of cancers. Prior studies focused on NLR, PLR and LMR showed these inflammatory biomarkers to be of prognostic value in GBC (9, 10). The prognostic significance of several inflammatory biomarkers were assessed in this study and SII performed significantly better than NLR, PLR and LMR. Furthermore, the inclusion of SII substantially improved the prognostic estimates in patients with GBC.

The relation between SII and bilirubin levels has been studied in pancreatic cancer (25). The current study supported the prognostic value of SII for GBC, as in pancreatic cancer, decreased with higher total bilirubin levels. To our knowledge, no prior study has evaluated the prognostic value of SII with CA19-9 levels and microvascular invasion (26). The current study showed SII to lose its prognostic value at high CA19-9 levels, indicating that SII has the potential to be a prognostic marker only in the setting of a CA19-9 level<40 U/ml. Thus, SII should be used with caution in the setting of high CA19-9 levels.

After radical cholecystectomy for GBC, long-term survival in an individual patient stratified by the TNM system can vary tremendously and is difficult to predict. This study sought to develop a nomogram by combining various factors in predicting long-term survival outcomes after radical cholecystectomy for GBC. This nomogram performed well in predicting survival, as supported by the C-indexes of 0.735 and 0.686 for the discovery and validation cohorts, respectively.

Researchers have been actively finding effective adjuvant therapies for GBC which has a high recurrence rate after radical cholecystectomy (27). However, the efficacy and benefit of postoperative chemotherapy in these patients have been controversial. Negative results were found in a randomized phase Ш trial for a gemcitabine-based regimen (28). In a systematic review and meta-analysis of patients with biliary tract cancer, Horgan et al. (29) reported an insignificant improvement in OS using adjuvant therapy for GBC patients when compared with surgery alone. Retrospective studies concluded that adjuvant chemotherapy improved OS in patients with T2N1 or T3N1 GBC with microscopic residual diseases (R1) (30–33). However, no definitive conclusion could be drawn on postoperative adjuvant therapy for patients with T2N0 or T3N0 GBC after radical cholecystectomy. The nomogram and the novel staging system in this study were able to identify patients with unfavorable long-term prognosis after radical cholecystectomy (the novel stages II, Ш for T2N0 and novel stages Ш, IV for T3N0). They can also be used as a predictive tool for pathological response to adjuvant chemotherapy in patients with GBC. Future studies on postoperative adjuvant therapy should be conducted on this group of patients.

Prior studies on nomograms for GBC have identified different significant factors in predicting long-term survival outcomes (34–37). These nomograms showed better prognostic values than the commonly used TNM staging system. However, very few of these nomograms used preoperative inflammatory indices for prediction. **Supplementary Table 4** summarizes the major studies on nomograms in predicting OS in GBC. In this study, models including and excluding SII were compared. The nomogram which combined inflammatory indices with pathological features performed better (0.735 versus 0.726, p<0.01), indicating that preoperative inflammatory laboratory tests should be included in the nomogram.

Incorporating prognostic biomarkers in clinical practice is challenging. SII cannot be used to select patients to undergo radical cholecystectomy, and surgical resection is still the only curative treatment for GBC patients. This study did not compare the differences in SII between surgical and non-surgical patients, and whether to perform operation was dependent on the TNM staging (21).However, it is still important to study preoperative biomarkers as recent evidences showed inflammatory biomarkers was associated with pathological response to neoadjuvant chemotherapy and immunotherapy (38–41) and the application of immune checkpoint inhibitors in treating various cancers (42) has made studies on immune-specific biomarkers even more important, The easy accessibility of SII makes it a potentially useful candidate biomarker for clinical studies on immunotherapy for GBC.

Surveillance, Epidemiology, and End Results (SEER) has been one of the most commonly used cancer registry database which included 13373 gallbladder cancer patients. However, preoperative blood tests of patients are not available. The Chinese Research Group of Gallbladder Cancer (CRGGC) Project is a national multicenter retrospective tumor registry which has collected from 2000 to 2019 data of more than 9496 patients, and the number is still increasing. This project contains preoperative laboratory tests in calculating inflammatory indices.

There are several limitations of this study. First, it is a retrospective study with its inherent defects, including limited availability of laboratory data at various preoperative time points. Second, some pathological features, including vascular invasion and nerve invasion, are not available. Third, as there are no universally accepted standards on the cut-off points used in converting continuous variables into category variables, this study calculated the cut-off point of SII based on statistical methods, and it was different from the cut-off points used for other cancers (25, 43). Whether the differences in the cut-off points used are due to different types of tumors or due to insufficient number of patients used in the studies are still unknown. Fourth, this study spanned over a relatively long period of time, and progress in surgical technology and techniques can affect OS to some extent. The validation cohort was divided into 2 groups as patients before 2014 and patients after 2014, and difference of predictive performance of the nomogram was noticed. The C-index in patients before 2014 was 0.661(95% CI:0.591-0.731) and in patients after 2014 was 0.709 (95% CI:0.627-0.791). Finally, external validation of these findings in other cohorts, which is essential, has not been done.

In conclusion, this study is a large study on the value of SII in patients with GBC after radical cholecystectomy. A nomogram was constructed by combining both preoperative and pathological features. This nomogram showed good accuracy in predicting long-term survival outcomes of patients with GBC after radical cholecystectomy; and the new staging system could be used to identify groups of lymph node negative patients with T2 and T3 GBC with unfavorable prognosis who could benefit from adjuvant therapy. Further prospective studies are needed to confirm the findings of this study.

## Data Availability Statement

The raw data supporting the conclusions of this article will be made available by the authors, without undue reservation.

## Ethics Statement

Ethical approval was obtained from the Ethics Committee of Renji Hospital Affiliated to Shanghai Jiao Tong University School of Medicine.

## Author Contributions

LL: Data curation, formal analysis, investigation, methodology, project administration, software, validation, visualization, writing – original draft, and writing – review and editing. TR: Methodology, project administration, software, validation, visualization, writing – original draft, and writing – review and editing. KL: Methodology, project administration, software, validation, visualization, and writing – original draft, writing – review and editing. M-LL: Data curation, formal analysis, validation, visualization, and writing – review and editing. YY: Methodology, software, visualization, and writing – review and editing. H-FL: Funding acquisition, resources, supervision, and writing – review and editing. X-CL: Methodology, software, visualization, and writing – review and editing. R-FB: Methodology, resources, and supervision. Y-JS: Resources and supervision. HW: Methodology, software, and visualization. WG: Project administration, resources, validation, and writing – review and editing. WL: Project administration, resources, validation, and writing – review and editing. X-SW: Conceptualization, funding acquisition, investigation, methodology, supervision, validation, and writing – review and editing. Y-BL: Conceptualization, funding acquisition, project administration, resources, supervision, and writing – review and editing. All authors contributed to the article and approved the submitted version.

## Funding

This study was supported by Shanghai Key Laboratory of Biliary Tract Disease Research Foundation(17DZ2260200), the National Natural Science Foundation of China (No. 81502433, 81773043, 91440203, 81702315), Clinical Research Program of Xinhua Hospital (19XHCR3D), Multi-center Clinical Research Project of Shanghai Jiaotong University School of Medicine(DLY201507), the Project of Excellent Young Scholars from Shanghai Municipal Health and Family Planning Commission (No. 2018YQ10), the Talent Development Fund from Shanghai Municipal Human Resources and Social Security Bureau (No. 2018048), and the Project of Experimental Animal Research from Science and Technology Commission Shanghai Municipality (No. 19140902700) and Shanghai Sailing Program (No.21YF1428700), Shanghai national science foundation (20ZR1435200) and Shanghai joint research projects on emerging frontier technologies (SHDC12018107).

## Conflict of Interest

The authors declare that the research was conducted in the absence of any commercial or financial relationships that could be construed as a potential conflict of interest.
