# Supplementary material for: Development and Validation of a Prognostic Nomogram Based on the Systemic Immune-Inflammation Index for Resectable Gallbladder Cancer to Predict Survival and Chemotherapy Benefit
Source: Front Oncol. 2021 Jun 29;11:692647. doi: 10.3389/fonc.2021.692647 (PMC8276054; doi:10.3389/fonc.2021.692647)
Supplement: Supplementary file 1 [file DataSheet_1.docx]

Supplementary Material

# Supplementary Figure


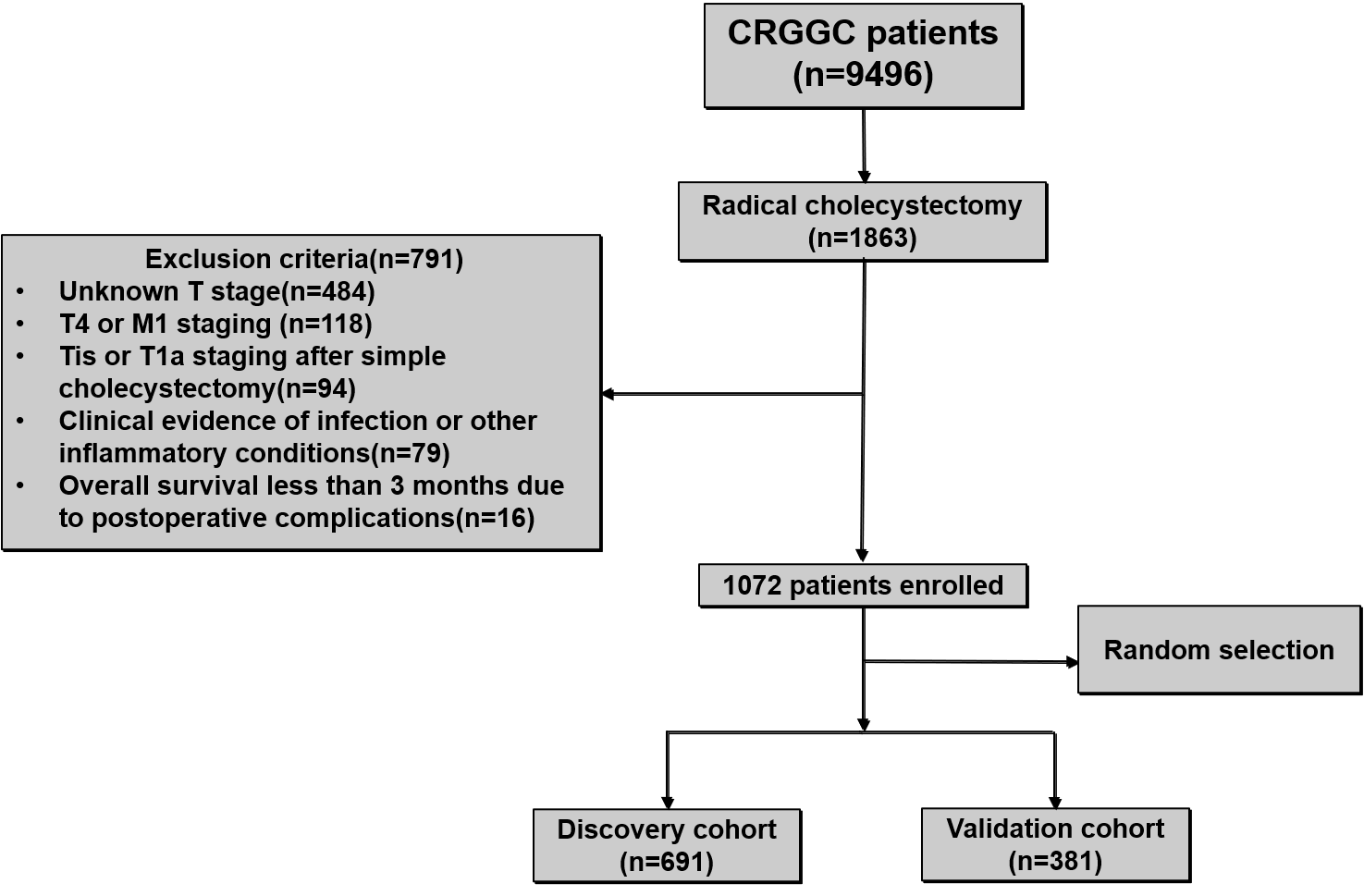


**Supplementary Figure 1.** Flowchart of the enrolled patients


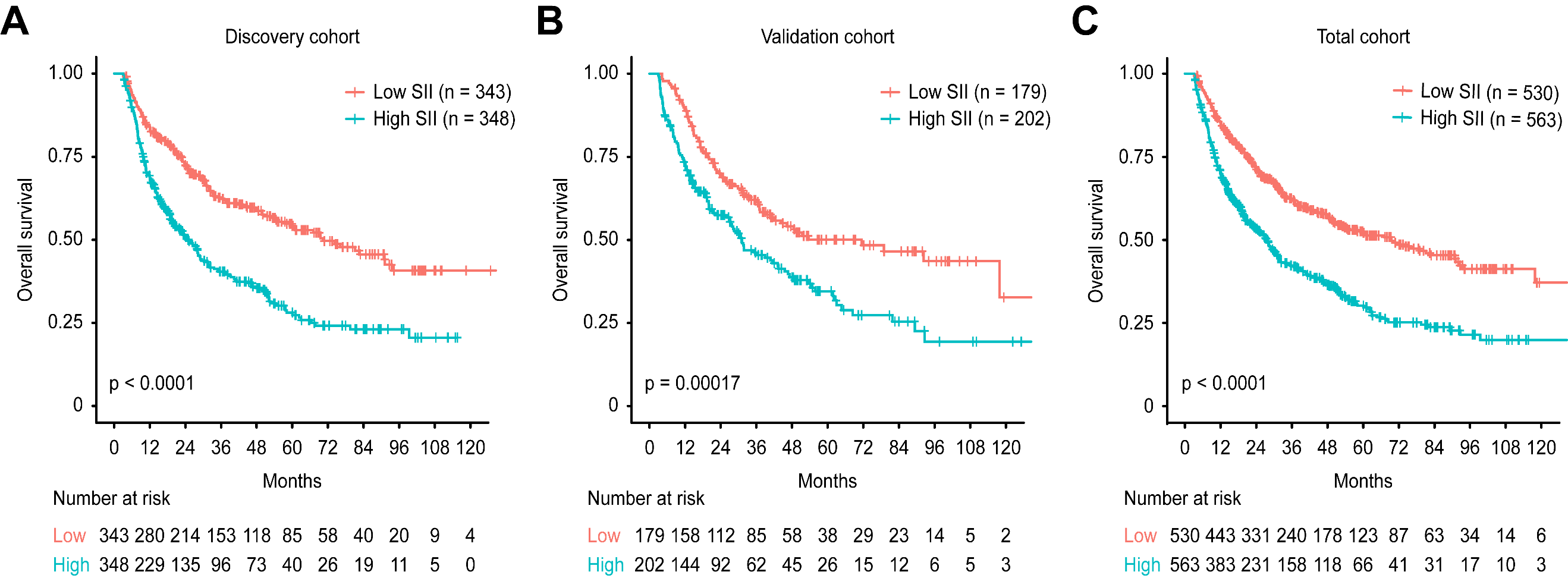


**Supplementary Figure 2.** Kaplan-Meier curves for SII in relation to OS in discovery(A), validation(B) and total cohorts(C)

**
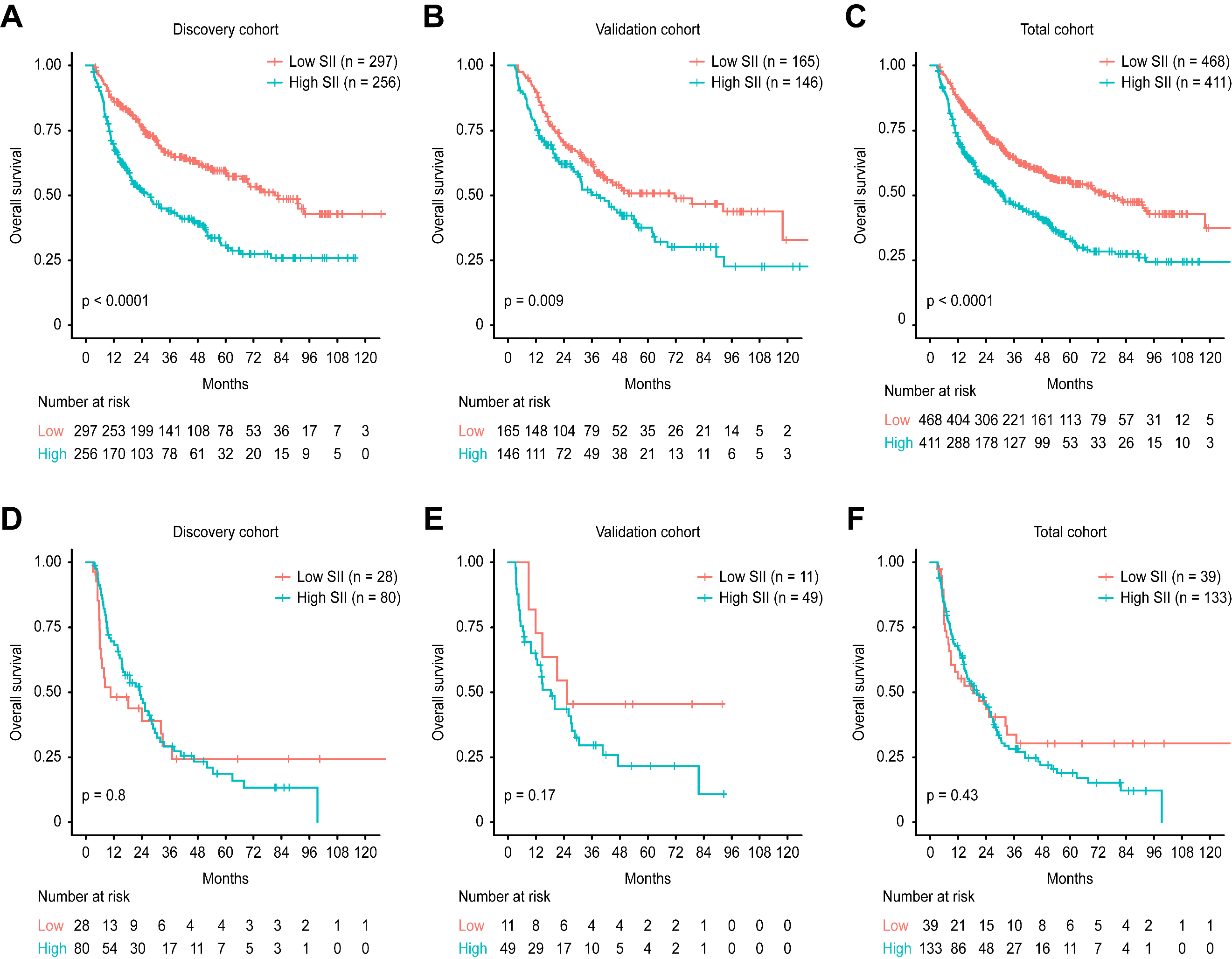
**

**Supplementary Figure 3.** Kaplan-Meier curves of OS for patients with high versus low SII in the setting of normal and high bilirubin levels, (A) discovery cohort with normal bilirubin (B) validation cohort with normal bilirubin (C) total cohort with normal bilirubin (D) discovery cohort with high bilirubin (>35µmol/L) (E) validation cohort with high bilirubin (>35µmol/L) (F) total cohort with high bilirubin (>35µmol/L)


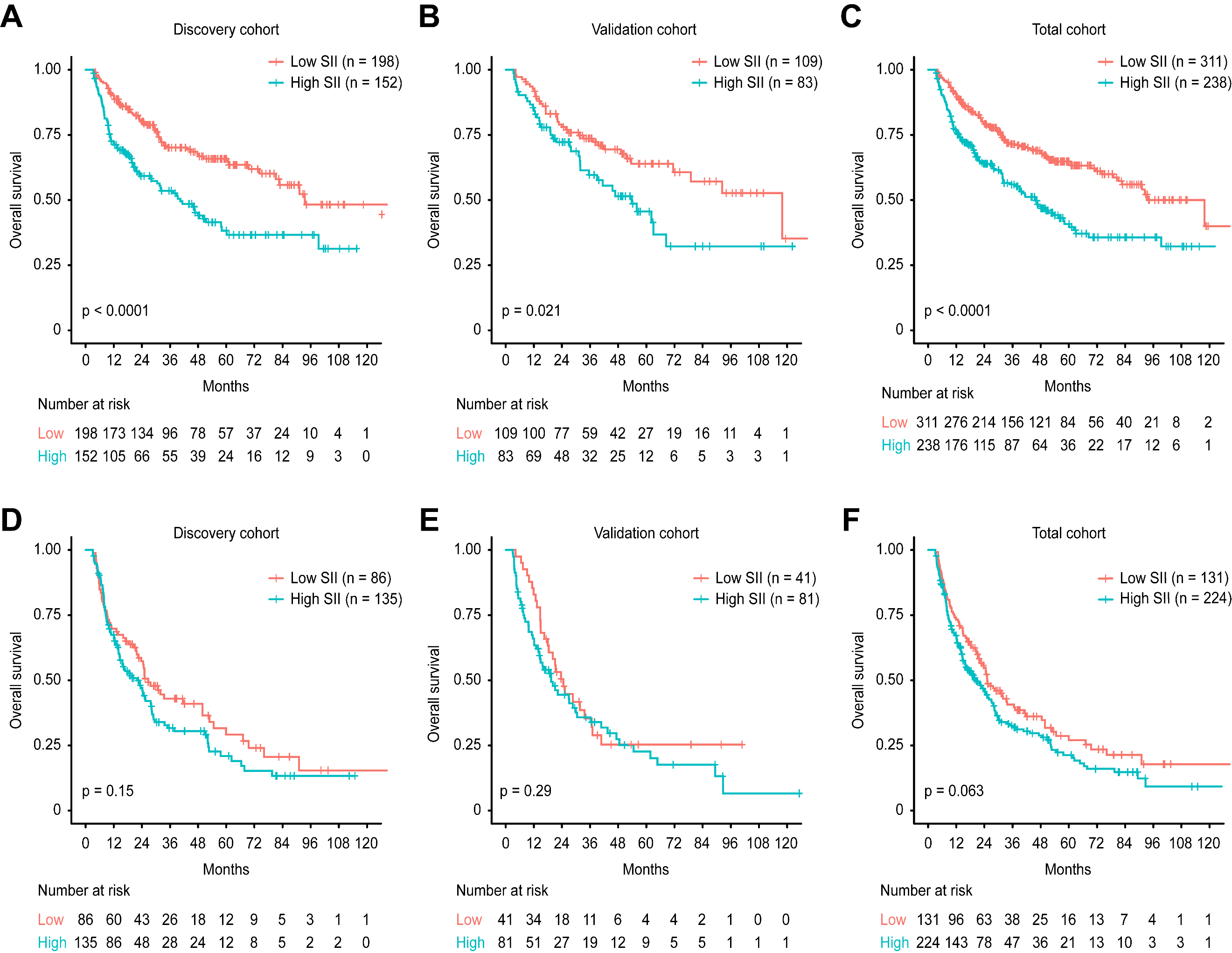


**Supplementary Figure 4.** Kaplan-Meier curves of OS for patients with high versus low SII in the set of low and high CA19-9 levels, (A) discovery cohort with low CA19-9 levels (B) validation cohort with low CA19-9 levels (C) total cohort with low CA19-9 levels (D) discovery cohort with high CA19-9 levels (>40U/ml) (E) validation cohort with high CA19-9 levels (>40U/ml) (F) total cohort with high CA19-9 levels (>40U/ml)


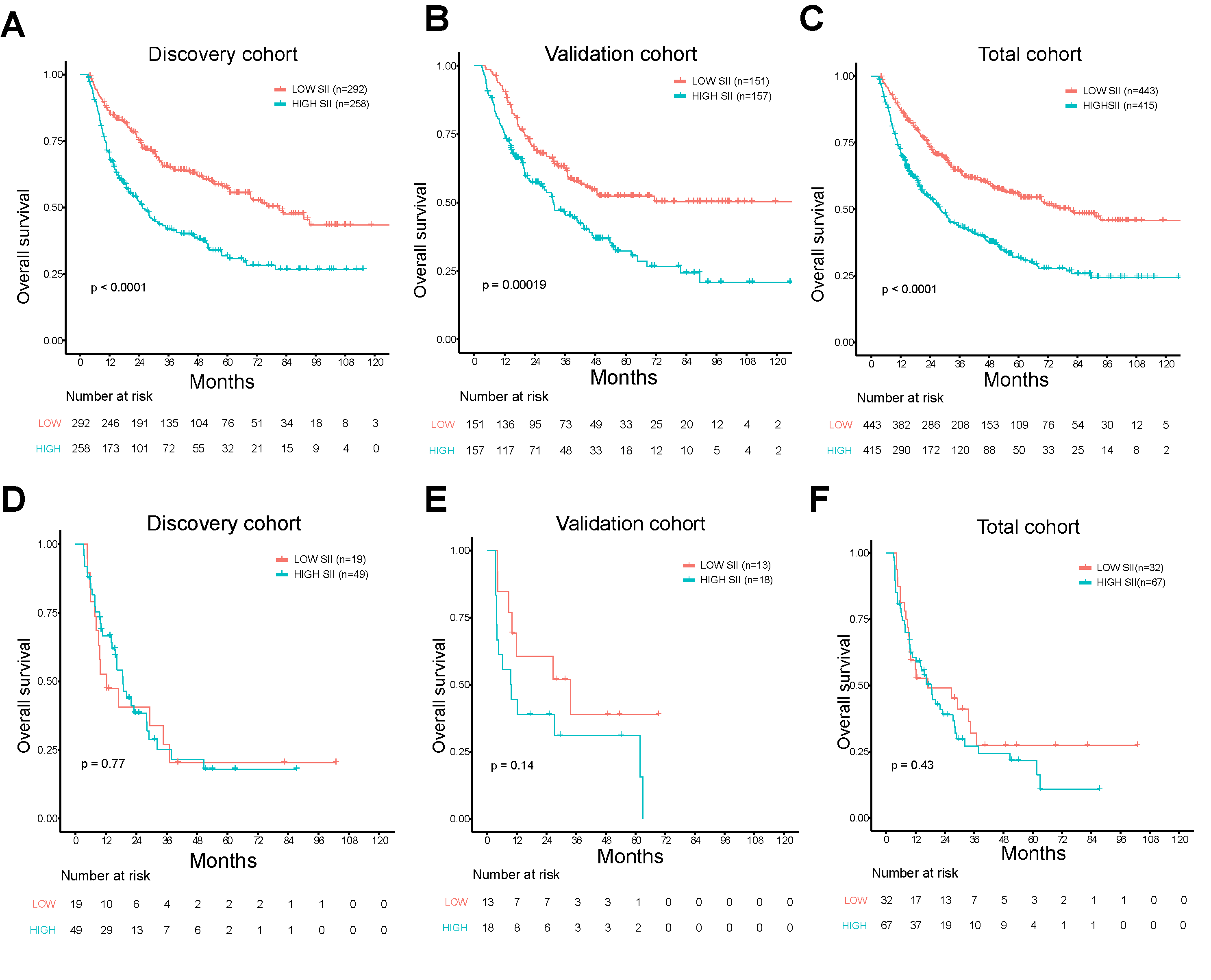


**Supplementary Figure 5.** Kaplan-Meier curves of OS for patients with high versus low SII. (A) discovery cohort without microvascular invasion (B) validation cohort without microvascular invasion (C) total cohort without microvascular invasion (D) discovery cohort with microvascular invasion (E) validation cohort with microvascular invasion (F) total cohort with microvascular invasion


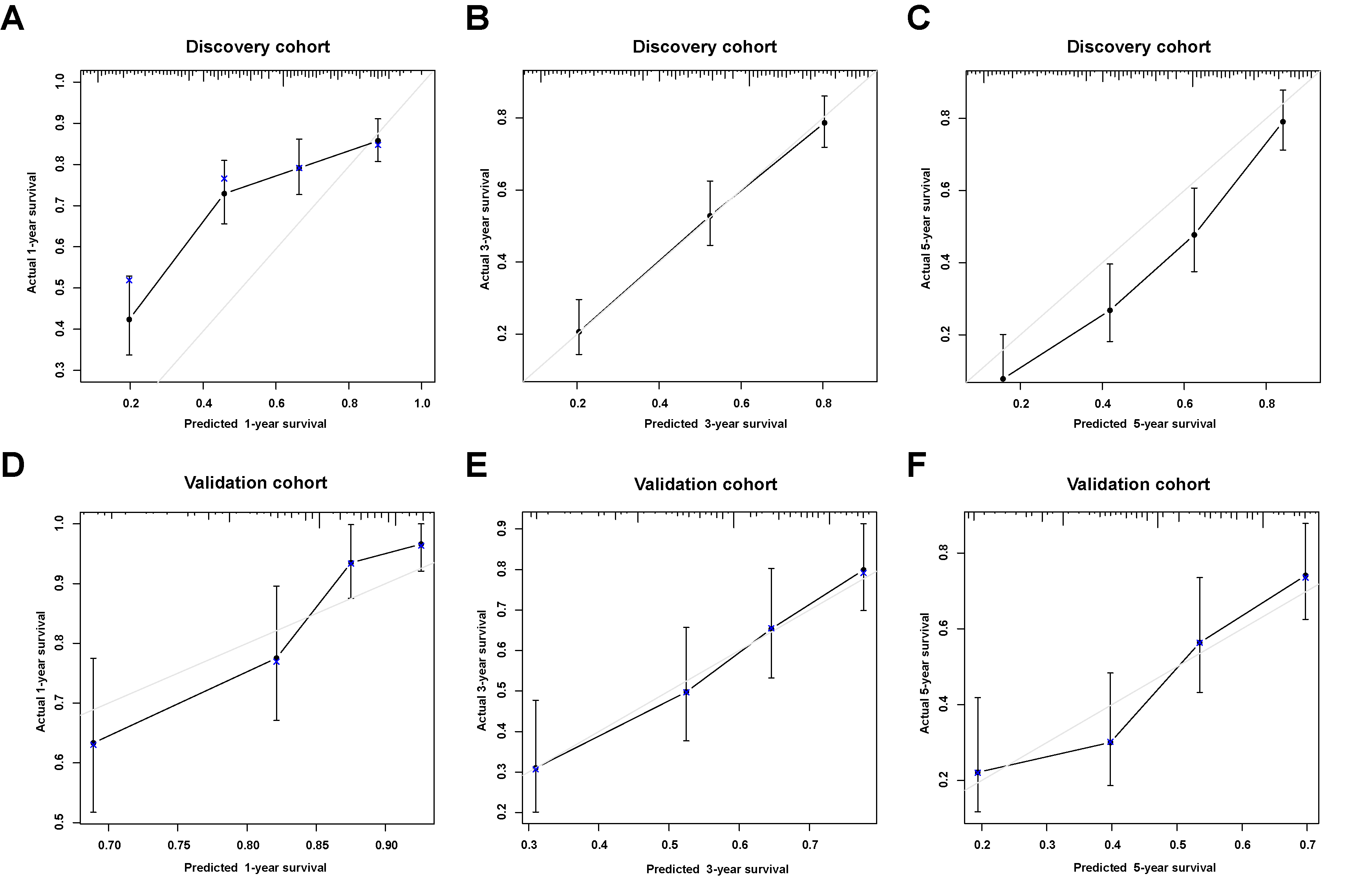


**Supplementary Figure. 6** The calibration curves for predicting survival probabilities of 1 (A), 3 (B) and 5 (C) year in the discovery cohort and of 1 (D), 3 (E) and 5 (F) year in the validation cohort


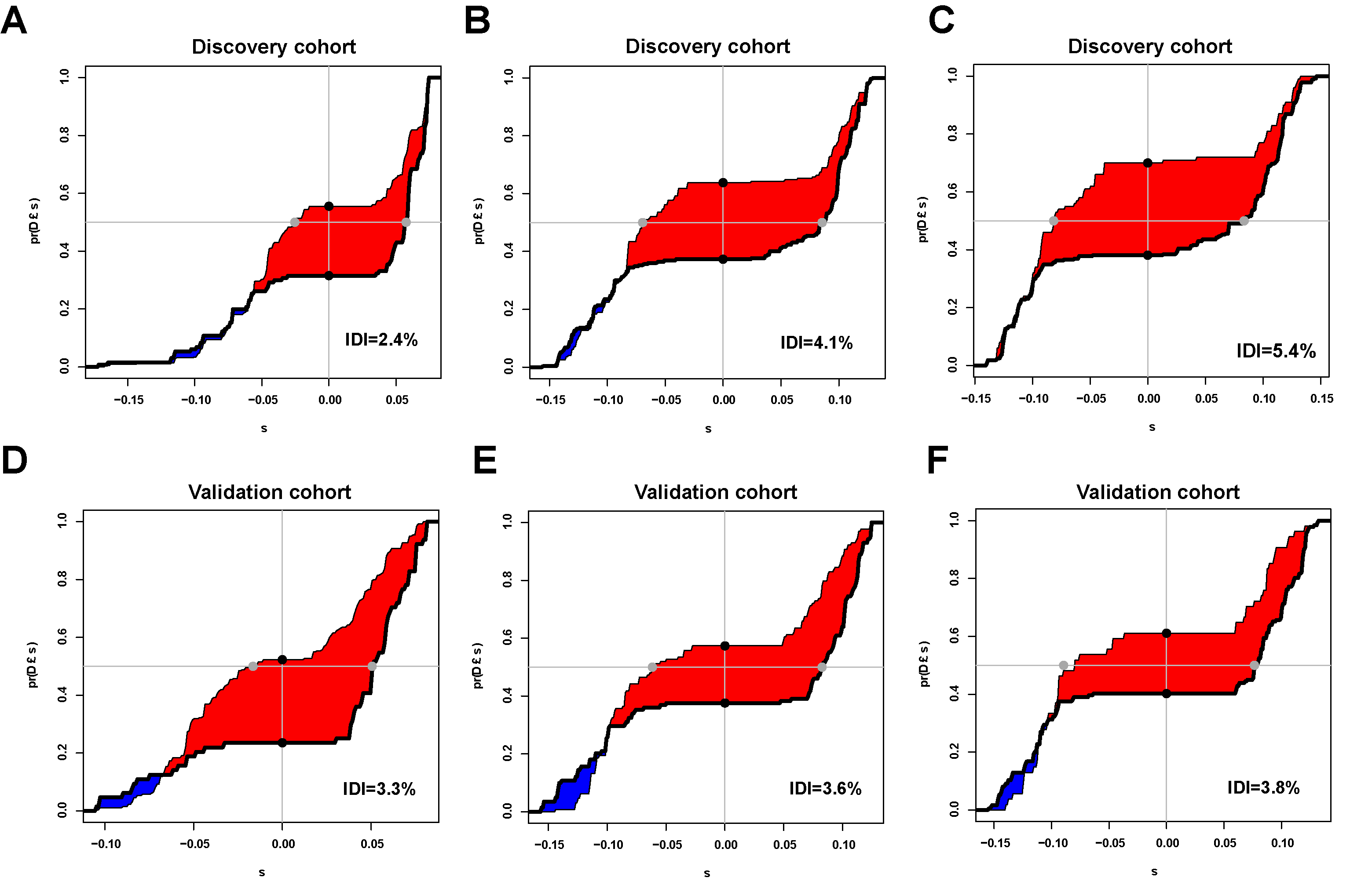


**Supplementary Figure. 7** The IDI curves for estimating predictive performance of 1 (A), 3 (B) and 5 (C) years in the discovery cohort and of 1 (D), 3 (E) and 5 (F) years in the validation cohort


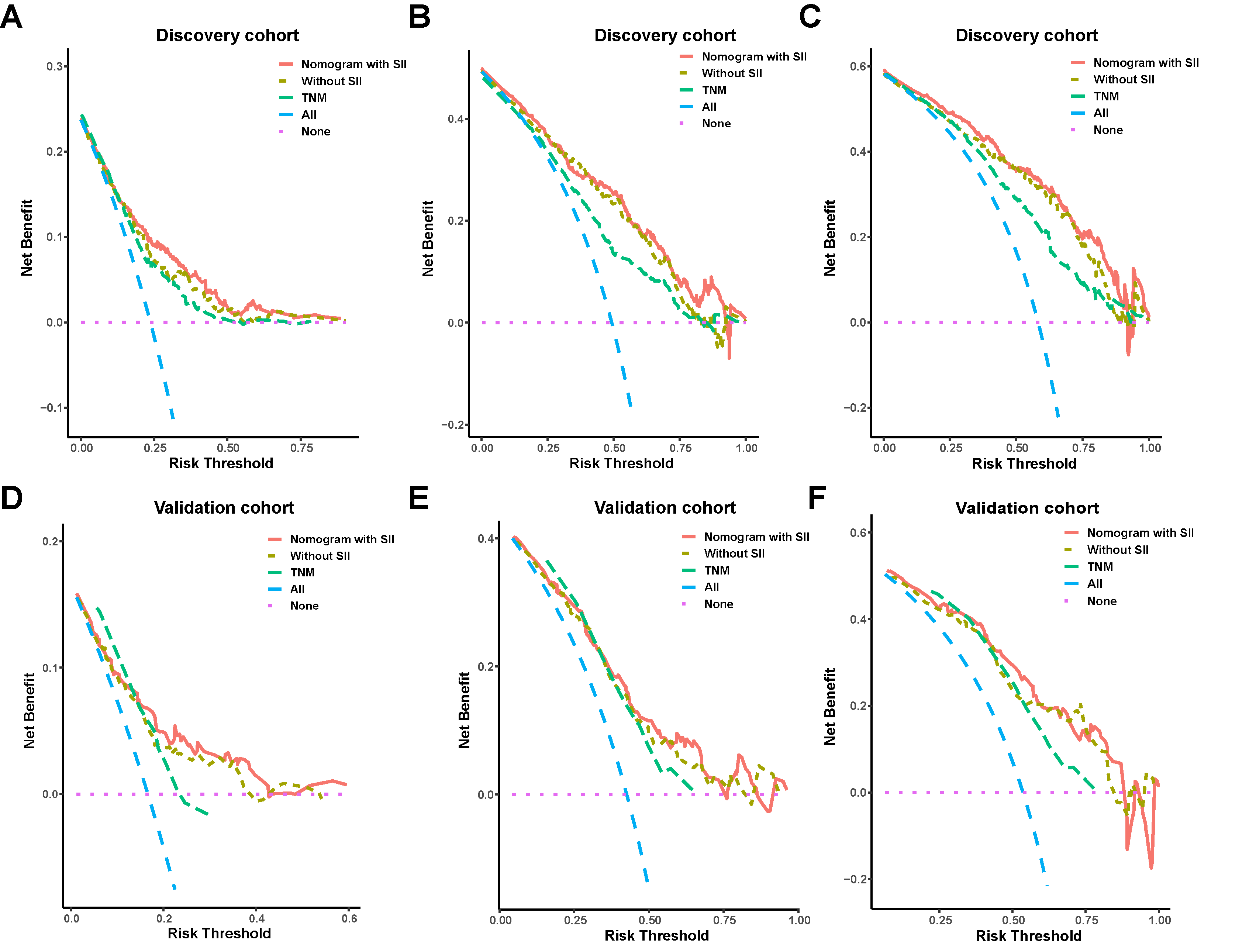


**Supplementary Figure. 8** Decision curve analysis presented the clinical net benefit of 1 (A), 3 (B) and 5 (C) years between nomogram, base model without SII and TNM staging system in the discovery cohort and 1 (A), 3 (B) and 5 (C) years in the validation cohort


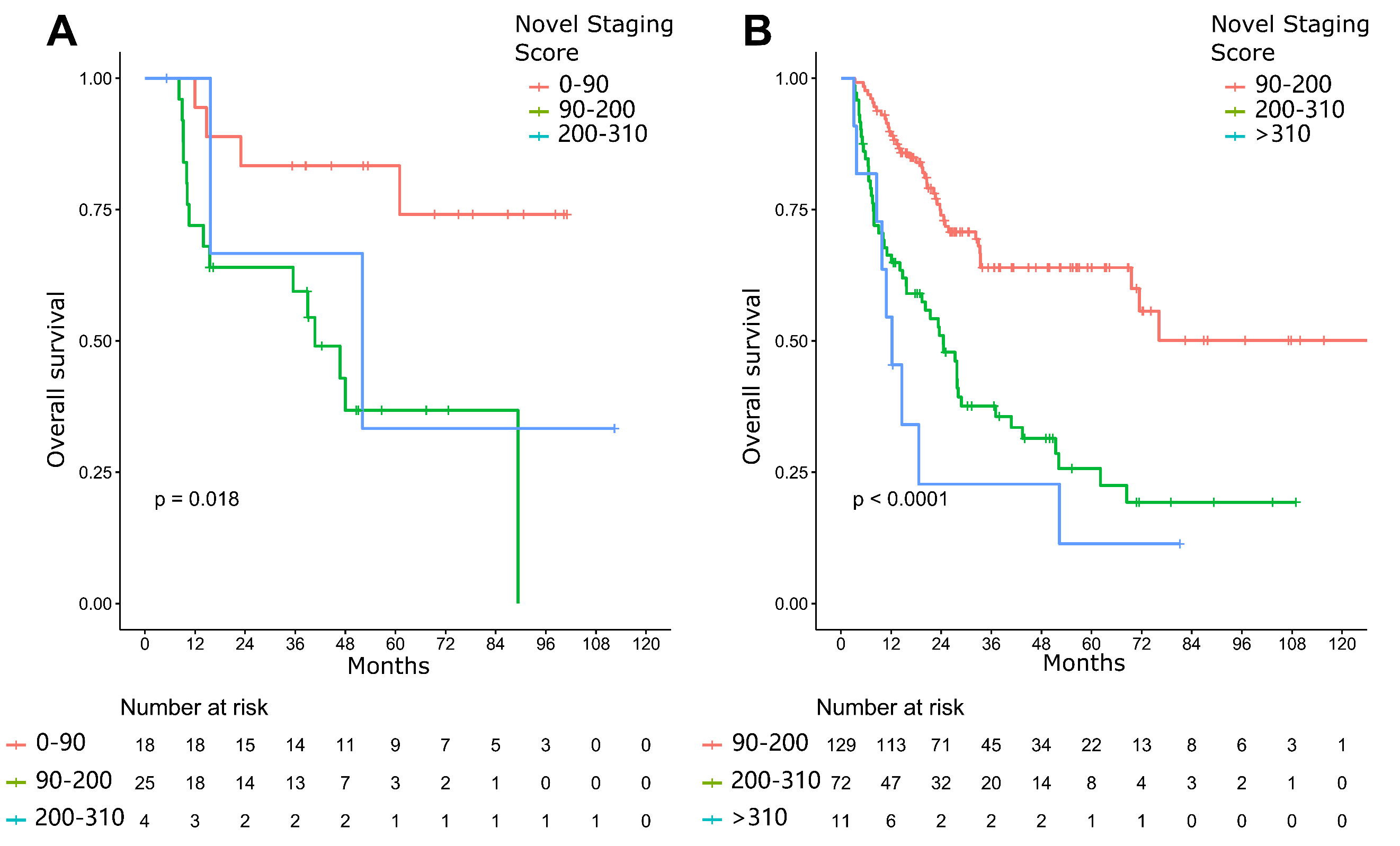


**Supplementary Figure 9.** Kaplan-Meier curves of OS for patients with T2N0 staging (A) and patients with T3N0 staging (B) Stratified using the Novel Staging System

# Supplementary Table

**Supplementary Table 1**. Multivariable Cox Regression Analysis Estimating Influence of Inflammatory Indices and Clinical Parameters on OS

|  |  | Base | Base+PLR | Base+NLR | Base+LMR | Base+SII | Base+SII*CA19-9 |
| --- | --- | --- | --- | --- | --- | --- | --- |
| CA19-9 |  |  |  |  |  |  |  |
|  |  | 1.78(1.33-2.38) | 1.74(1.30-2.33) | 1.67(1.24-2.23) | 1.77(1.32-2.37) | 1.67 (1.25-2.24) | 2.03(0.78-5.26) |
| Tumor differentiation |  |  |  |  |  |  |  |
| Low to medium |  | 0.61(0.41-0.93) | 0.63(0.41-0.95) | 0.62(0.41-0.94) | 0.64(0.42-0.97) | 0.63(0.42-0.96) | 0.63(0.42-0.96) |
| Medium |  | 0.54(0.38-0.76) | 0.55(0.39-0.77) | 0.54(0.38-0.76) | 0.53(0.37-0.75) | 0.53(0.37-0.75) | 0.53(0.38-0.76) |
| Medium to high |  | 0.42(0.21-0.84) | 0.43(0.22-0.87) | 0.42(0.21-0.84) | 0.46(0.23-0.91) | 0.42(0.21-0.84) | 0.42(0.21-0.85) |
| High |  | 0.34(0.16-0.69) | 0.35(0.17-0.72) | 0.36(0.17-0.75) | 0.36(0.17-0.74) | 0.40(0.19-0.83) | 0.40(0.19-0.82) |
| Margin |  |  |  |  |  |  |  |
|  |  | 1.75(1.13-2.70) | 1.66(1.08-2.56) | 1.64(1.07-2.54) | 1.71(1.11-2.64) | 1.55(1.01-2.39) | 1.54(1.00-2.38) |
| T stage |  |  |  |  |  |  |  |
| T2 |  | 1.29(0.62-2.67) | 1.31(0.63-2.73) | 1.45(0.69-3.04) | 1.42(0.68-2.97) | 1.39(0.66-2.92) | 1.39(0.66-2.93) |
| T3 |  | 2.30(1.19-4.46) | 2.36(1.21-4.60) | 2.50(1.27-4.92) | 2.33(1.20-4.54) | 2.58(1.31-5.10) | 2.58(1.31-5.09) |
| N stage |  |  |  |  |  |  |  |
| N1 |  | 1.09(0.75-1.57) | 1.08(0.75-1.56) | 1.16(0.80-1.67) | 1.21(0.83-1.77) | 1.11(0.77-1.59) | 1.10(0.76-1.59) |
| N2 |  | 2.36(1.38-4.03) | 2.45(1.43-4.20) | 2.45(1.43-4.21) | 2.54(1.48-4.36) | 2.43(1.42-4.17) | 2.42(1.41-4.15) |
| N9 |  | 1.43(1.00-2.06) | 1.47(1.02-2.12) | 1.46(1.01-2.10) | 1.52(1.05-2.19) | 1.40(0.97-2.01) | 1.39(0.97-2.00) |
| PLR |  |  |  |  |  |  |  |
| >144 versus ≤144 |  |  | 1.39(1.05-1.84) |  |  |  |  |
| NLR |  |  |  |  |  |  |  |
| >2.3 versus ≤2.3 |  |  |  | 1.70(1.27-2.28) |  |  |  |
| LMR |  |  |  |  |  |  |  |
| >10 versus ≤10 |  |  |  |  | 1.46(1.08-1.96) |  |  |
| SII |  |  |  |  |  |  |  |
| >510 versus ≤510 |  |  |  |  |  | 1.90(1.42-2.54) | 2.28(0.93-5.61) |
| SII*CA19-9 |  |  |  |  |  |  |  |
|  |  |  |  |  |  |  | 0.88(0.50-1.55) |
| C-index |  | 0.726 | 0.732 | 0.732 | 0.722 | 0.735 | 0.735 |
| AIC |  | 1267 | 1263 | 1255 | 1262 | 1249 | 1251 |

Crude model without inflammatory indices included following predictors as candidates: pre-operative CA19-9, resection margin, tumor differentiation, and T stage and N stage.

Abbreviation: AIC, Akaike information criterion.

**Supplementary Table 2.** Comparison of clinical characteristics of patients with high and low SII

|  | SII ≤510  （n=343） | SII >510  （n=348） | P value |
| --- | --- | --- | --- |
| Age^†^ | 62（57-69） | 63（55.7-70） | 0.72 |
| Sex |  |  | 0.75 |
| Male | 136 | 134 |  |
| Female | 207 | 214 |  |
| CA19-9 |  |  | <0.001 |
| ≤40 U/ml | 198 | 152 |  |
| >40 U/m | 86 | 135 |  |
| NA | 59 | 61 |  |
| Surgical approach |  |  | 0.09 |
| RC | 214 | 191 |  |
| ERC | 14 | 24 |  |
| LC+RC | 63 | 64 |  |
| NA | 52 | 69 |  |
| Total bilirubin |  |  | <0.001 |
| ≤35 μmol/L | 297 | 156 |  |
| >35 μmol/L | 28 | 80 |  |
| NA | 18 | 12 |  |
| Margin status |  |  | 0.003 |
| R0 | 21 | 44 |  |
| R1 | 293 | 274 |  |
| Rx | 29 | 30 |  |
| Pathological type |  |  | 0.14 |
| ADC | 293 | 276 |  |
| ADSC | 12 | 23 |  |
| PADC | 14 | 13 |  |
| NEC | 5 | 5 |  |
| Other | 19 | 31 |  |
| Tumor differentiation |  |  | 0.004 |
| Low | 58 | 87 |  |
| Low to medium | 56 | 62 |  |
| Medium | 127 | 129 |  |
| Medium to high | 24 | 16 |  |
| High | 43 | 19 |  |
| NA | 35 | 35 |  |
| T stage^‡^ |  |  | 0.02 |
| T1 | 56 | 35 |  |
| T2 | 41 | 33 |  |
| T3 | 246 | 280 |  |
| N stage^‡^ |  |  | 0.07 |
| N0 | 168 | 136 |  |
| N1 | 70 | 89 |  |
| N2 | 18 | 23 |  |
| Nx | 87 | 100 |  |
| Microvascular invasion |  |  | 0.002 |
| Yes | 19 | 49 |  |
| No | 292 | 258 |  |
| NA | 32 | 41 |  |
| Perineural invasion |  |  | <0.001 |
| Yes | 39 | 79 |  |
| No | 280 | 239 |  |
| NA | 24 | 30 |  |
| Platelets |  |  | <0.001 |
| ≤300*10^^9^/L | 332 | 259 |  |
| >300*10^^9^/L | 11 | 89 |  |

† Age is presented as the median (first quartile-third quartile).

‡ T and N stage classification according to the AJCC 8^th^ edition staging system.

Abbreviations: ADC, adenocarcinoma; ADSC, adenosquamous carcinoma;

PADC, papillary adenocarcinoma; NEC, neuroendocrine carcinoma;

RC, radical resection; ERC, extended radical resection;

LC, laparoscopic cholecystectomy; RC, radical cholecystectomy;

ERC, extended radical cholecystectomy;

NLR, neutrophil-to-lymphocyte ratio; PLR, platelet-to-lymphocyte ratio;

LMR, lymphocyte-to-monocyte ratio; SII, systemic immune-inflammation index.

**\**

**Supplementary Table 3.** Characteristics of the T2N0 or T3N0 Patients Stratified by the Novel Staging System

|  | Good prognosis | Poor prognosis |
| --- | --- | --- |
| CA19-9 |  |  |
| ≤40 U/ml | 12 | 41 |
| >40 U/ml | 80 | 27 |
| Tumor differentiation |  |  |
| Low | 7 | 21 |
| Low to medium | 9 | 20 |
| Medium | 62 | 22 |
| Medium to high | 4 | 1 |
| High | 10 | 4 |
| T stage |  |  |
| T2 | 21 | 19 |
| T3 | 71 | 49 |
| SII |  |  |
| ≤510 | 75 | 8 |
| >510 | 17 | 60 |
| Adjuvant chemotherapy | 41 | 26 |
| GEMOX+5-FU | 4 | 3 |
| GEMOX | 10 | 7 |
| GEM+5-FU | 7 | 3 |
| GEM | 7 | 6 |
| Folfirinox | 1 | 0 |
| Capecitabine | 1 | 2 |
| Irinotecan | 1 | 1 |
| 5FU+CF/Oxaliplatin | 4 | 1 |
| EP | 3 | 1 |
| Unknown | 3 | 2 |

Abbreviations: GEM, gemcitabine; GEMOX, gemcitabine plus oxaliplatin;

5-FU, 5-Fluorouracil; CF, calcium folinate; EP, etoposide plus cis-platinum

**Supplementary Table 4.** Comparison of Major Studies of Nomograms in GBC

|  | | | | |
| --- | --- | --- | --- | --- |
|  | Type of study | Sample size | Parameters | Significance of study |
| Wang et al. | SEER database | 4180 | Age, sex, race, histology | Determined the role of adjuvant radiation therapy in resected GBC |
| Wang et al. | SEER database | 1137 | Age, sex, race, T stage, N stage | Determined the role of adjuvant chemotherapy and chemoradiation in resected GBC |
| Zhang et al. | SEER database | 1422 | Age, sex, lymph node dissection,  tumor size, tumor differentiation,  T stage, N stage, postoperative chemotherapy | Predicted the prognosis of resected GBC |
| Bai et al. | Single-center study | 142 | Jaundice, CA19-9, tumor stage, margin status | Predicted the prognosis of resected GBC |
| Yadav et al. | Tertiary care center | 528 | Age, ECOG, tumor size, ALP,  metastatic disease | New staging system based on  preoperative parameters to determine OS |
| Sun et al. | Single-center study | 142 | CA19-9, TNM staging system, SII | Included inflammatory indices to achieve better prediction of OS in resected GBC |
| Current study | CRGGC program | 1093 | CA19-9, margin status, T stage,  N stage, tumor differentiation, SII | Included SII to predict chemotherapy response for T2N0 or T3N0 patients |

Abbreviations: SEER, Surveillance, Epidemiology, and End Results; ALP, alkaline phosphatase; ECOG, Eastern Cooperative Oncology Group Performance Status.
